# Severe Physical Child Abuse Presenting as Septic Shock: A Case Report of Traumatic Hemoperitoneum

**DOI:** 10.1155/crpe/5615796

**Published:** 2025-09-25

**Authors:** Madalena Carvalho, Catarina Nunes, Rita Martins, Mariana Silva, Carlos Escobar, Helena Isabel Almeida

**Affiliations:** ^1^Pediatrics Service, Department of Child, Hospital Professor Doutor Fernando Fonseca, Amadora, Portugal; ^2^Pediatric Intensive Care Unit, Pediatrics Service, Department of Child, Hospital Professor Doutor Fernando Fonseca, Amadora, Portugal; ^3^Social Pediatrics Team, Department of Child, Hospital Professor Doutor Fernando Fonseca, Amadora, Portugal

## Abstract

Child maltreatment refers to the abuse and neglect of children under the age of 18 and is a prevalent social problem that often goes undetected. To emphasize the importance of this diagnosis, we present the case of a 3-year-old boy who presented in shock with altered consciousness, initially managed as sepsis, but was ultimately diagnosed with severe physical abuse after imaging revealed a traumatic hemoperitoneum. With this article, we aim to remind healthcare providers to consider this diagnosis, even in severely ill children.

## 1. Introduction

Child and adolescent abuse represents a serious global concern that manifests across diverse social, ethnic, and racial groups [1]. According to the UNICEF estimates, nearly 400 million children under 5 years (6 in 10 children within this age group globally) regularly suffer physical punishment and/or psychological violence at the hands of parents and caregivers [2].

The term “child abuse” is a broad category encompassing a variety of forms of maltreatment, including physical abuse, sexual abuse, emotional abuse, and child neglect [1]. Child physical abuse is defined as nonaccidental injury (including bruises, cuts, burns, fractures, or other tissue damage) to the child inflicted by a parent or a caregiver in a parenting role [3].

The evaluation of suspected child abuse includes a complete history, a detailed physical examination and observation of interactions between parents/carers and the child. The identification of suspected abuse at the initial evaluation is critical in order to facilitate the treatment of injuries and to ensure the protection of all the children in the family from the possibility of more severe injury in the future. Nevertheless, the diagnosis of child maltreatment can often be challenging, with such cases frequently remaining undetected and underreported [4]. Many children are not diagnosed in the early stages of evaluation [5].

To emphasize the importance of this entity, we present a case of a 3-year-old boy admitted to the emergency room due to altered consciousness and scattered hematomas. The publication of the clinical case was approved by the ethics committee.

## 2. Case Report

A three-year-old male child, with no significant personal history was admitted to the emergency room due to an altered state of consciousness. He was accompanied by his mother and maternal grandmother. The mother reported an unquantified fever lasting 2 days, accompanied by diarrhea and vomiting. In the 5 days prior to admission, the patient was not in the care of his mother; he stayed with a family member together with his newborn sibling. Two days prior to admission, the mother made a visit to the child and observed two contusions on the face (resulting from a “fall from the bed”). The child had no headaches or changes in behavior. On the day of admission, when the mother visited the child, he exhibited minimal responsiveness, producing only barely perceptible sounds, and had disseminated skin hematomas.

In the emergency room, the patient was unresponsive to painful stimuli (Glasgow Coma Scale 5) and cold (tympanic temperature unmeasurable). There was poor peripheral perfusion, with scattered bruises across the face, trunk, and limbs. Cardiopulmonary auscultation and abdominal palpation were normal. There were no meningeal signs. The first recording of vital signs with oxygen saturation was 87% and heart rate was 150 beats per minute. Capillary blood glucose was measured at 203 mg/dL. A high-flow mask with oxygen was applied, and an intraosseous access was established in the proximal right tibia (after multiple unsuccessful attempts at peripheral access). The patient received a 10-mL/kg bolus of a balanced solution and was started on empirical antibiotic therapy following blood culture collection. Due to hypertonia followed by clonic movements of the limbs, rectal diazepam was administered. Initial blood gas analysis revealed metabolic acidosis and hyperlactatemia (Table 1).

After initial stabilization, the patient was transferred to the Pediatric Intensive Care Unit, where the patient was intubated and placed in mechanical ventilation. Due to persistent hypotension, an additional 10-mL/kg bolus was repeated followed by adrenaline infusion (0.1 mcg/kg/min), with arterial pressure improvement.

Laboratory results revealed (Table 1) anemia and elevated liver enzymes, without an increase in inflammatory markers. Other laboratory results showed no significant changes, and the chest X-ray was unremarkable. The patient was evaluated by Pediatric Cardiology upon admission; echocardiography showed normal cardiac function and no pericardial effusion. Urine analysis showed hematuria, without other abnormalities, and a CT scan of the head showed no significant findings. Arterial blood gas 2 hours after admission showed resolution of metabolic acidosis and hyperlactacidaemia.

During the first hours of hospitalization, the patient had fever (38°C). A lumbar puncture was performed, with no abnormalities. Toxicology testing was carried out on blood and urine samples, and all results were negative. The patient demonstrated progressive clinical improvement and neurological recovery within a few hours. The patient was extubated 6 hours after admission. After extubation, the child was alert and responsive, with a normal neurological examination.

**Figure 1:**
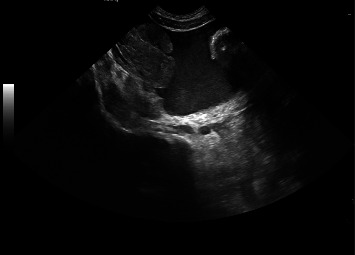
Abdominal ultrasound with hemoperitoneum.

**Figure 2:**
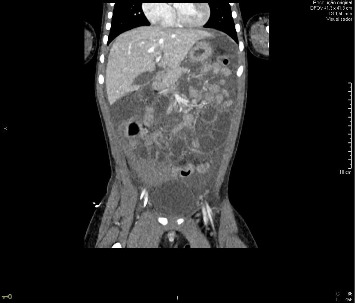
Abdominal computed tomography scan.

Twenty four hours after admission, the patient started with abdominal pain and had a fall in hemoglobin (5.5 g/dL), without overt bleeding. A red blood cell concentrate transfusion was performed. An abdominal ultrasound and a computed tomography scan revealed “a large amount of free intraperitoneal fluid with nonwater densities, consistent with hemoperitoneum; a large clot in the pelvic cavity with hematic densities, lateralized to the right; no pneumoperitoneum; some loops of the small intestine with increased caliber, of undetermined etiology; liver, pancreas, spleen, and kidneys without abnormalities” (Figures 1 and 2). In this context, the patient was evaluated by Pediatric Surgery. After reviewing the abdominal CT scan images, it was concluded that the patient had hemoperitoneum originating from a vascular injury of the mesentery (an injury compatible with high-kinetic traumatic mechanism). A conservative management was decided.

The presence of scattered ecchymoses and hemoperitoneum strongly suggested child abuse. The child abuse response protocol was activated, and the case was evaluated by the Social Pediatrics Team and referred to protection authorities and law enforcement. A suspect was put into custody and ultimately admitted to repeatedly beat and kick the child the days before admission.

A complete skeletal survey was performed as part of the child abuse evaluation, and no radiographic evidence of acute or healing fractures was identified. There were no significant complications during the remainder of the patient's hospital stay. The child was discharged on the 10th day of hospitalization.

## 3. Discussion

Physical child abuse should be considered for all pediatric patients who refer to the emergency department with unexplained conditions [5]. The manifestations of physical child abuse can vary widely, with several factors recognized as potential indicators [5]. Signs and symptoms of child abuse often include unexplained head and dental injuries, soft‐tissue injuries such as bruises and bite marks, or burns and fractures such as broken ribs, in the absence of a history or rational explanation for the cause of the trauma [1].

The diagnosis of child maltreatment can be challenging. In the case reported, the diagnosis of physical abuse was not the first hypothesis considered. The scattered ecchymoses, the history of gastrointestinal symptoms, and fever allied with the severity of the clinical presentation led the medical team to consider the hypothesis of sepsis as the primary diagnosis, which can be accompanied by hemorrhagic dyscrasia (petechiae and purpura) [6, 7]. The initial metabolic acidosis and cardiovascular compromise also pointed to this diagnosis. Therefore, in addition to fluid therapy, antibiotic therapy was initiated in the emergency room, following the golden hour guidelines. While the initial presentation of shock and metabolic acidosis was highly suggestive of sepsis, several key findings argued against it. The C-reactive protein was not significantly elevated, and although the procalcitonin level was slightly raised (1.88 ng/mL), it was unexpectedly low for a patient presenting with septic shock. Moreover, the patient's metabolic derangements and shock state resolved remarkably quickly following minimal fluid resuscitation, a clinical course less typical for severe bacterial sepsis. The results of the abdominal CT scan, in conjunction with the presence of scattered hematomas, indicated the hypothesis of physical abuse. A thorough review of the clinical history was also important to clarify the situation.

While no child is responsible for their experiences of abuse or neglect, some child characteristics have been identified as potentially increasing the risk of maltreatment. These characteristics include age and the presence of special healthcare needs or disabilities [8]. In the case presented, the child belonged to a high-risk age group (under 4 years) [9], was taken care together with his newborn brother by another family member, and maternal presence was scarce.

The management of child abuse can be difficult and often requires a multidisciplinary approach. This includes professionals who identify the cause of the abuse or neglect, treat the immediate problems, and refer the child to the appropriate child protection authority for intervention [1]. In this case, the collaboration with the Pediatric Surgical Team and Social Pediatrics Team was essential in the management and follow-up of the case.

Child maltreatment represents a significant public health concern, with impairments in behavioral and physiological functioning across the lifespan [3]. The early recognition and intervention in cases of child abuse is crucial, as early life adversity is a major risk factor for the development of psychopathology [10]. Among young children, there is an association between child maltreatment and lower cognitive skills, anxious, depressed, aggressive behaviors and poor emotional, social, and school functioning [8]. In adolescence and adulthood, child maltreatment is associated with poor mental health, problematic substance use behaviors, and chronic conditions such as asthma, diabetes, pain, and obesity [8].

## 4. Conclusion

Children who are victims of abuse may present with severe clinical conditions, including shock. It is important to consider child abuse when the clinical presentation is atypical or inconsistent, including seriously ill children. With this article, we aim to remind healthcare providers to consider this diagnosis.

## Figures and Tables

**Table 1:** Gasometry and blood tests (admission).

*Blood gas*	
pH	7 (RV 7.35–7.45)
CO_2_	39.5 mmHg (RV 35–45 mmHg)
HCO_3_-	9.3 mmol/L (RV 22–26 mmol/L)
Base excess	−20
Potassium	4.6 mmol/L
Sodium	139 mmol/L
Ionized calcium	1.29 mmol/L
Lactate	20 mmol/L (VR < 1.8 mmol/L)
Glucose	77 mg/dL

*Complete blood count*	
Hemoglobin	8.4 g/dL (RV 11–14 g/dL)
Leucocytes	13,700 cells/μL (RV 5000–150,000 cells/μL)
Platelets	326,000 cells/μL (RV 200,000–490,000 cells/μL)
*Renal/metabolic panel*	
Creatinine	0.74 mg/dL (RV 0.31–0.47 mg/dL)
Urea	48 mg/dL (RV 15–36 mg/dL)
Sodium	138 mmol/L (RV 138–145 mmol/L)
Potassium	4.8 mmol/L (RV 3.4–4.7 mmol/L)
Chlorine	96 mmol/L (RV 98–107 mmol/L)
Lactate dehydrogenase	1525 U/L (RV 120–300 U/L)
Glucose	179 mg/dL (RV 74–109 mg/dL)
C-reactive protein	0.61 mg/dL (RV < 0.5 mg/dL)
Procalcitonin	1.88 ng/mL
Albumin	3.88 mg/dL (RV 3.8–5.4 g/dL)

*Liver tests*	
Aspartate aminotransferase	439 U/L (RV < 48 U/L)
Alanine aminotransferase	217 U/L (RV < 29 U/L)
Alkaline phosphatase	203 U/L (RV 129–417 U/L)
Gamma glutamyl transferase	19 U/L (RV < 26 U/L)

*Coagulation*	
Prothrombin time	12.7 s (RV 9.7–11.8 s)
Activated partial thromboplastin time	21.6 s (RV 23–31.9 s)

## Data Availability

The data that support the findings of this study are available from the corresponding author upon reasonable request.
